# A coproduced patient and public event: An approach to developing and prioritizing ambulance performance measures

**DOI:** 10.1111/hex.12606

**Published:** 2017-08-25

**Authors:** Andy Irving, Janette Turner, Maggie Marsh, Andrea Broadway‐Parkinson, Dan Fall, Joanne Coster, A. Niroshan Siriwardena

**Affiliations:** ^1^ School of Health and Related Research (ScHARR) University of Sheffield Sheffield UK; ^2^ Sheffield Emergency Care Forum Sheffield UK; ^3^ ‘Dispel’ Disability Consultancy York UK; ^4^ School of Health and Social Care University of Lincoln Community and Health Research Unit Christchurch New Zealand

**Keywords:** Ambulance service, co‐production, health service research, patient and public involvement, pre‐hospital health care

## Abstract

**Background:**

Patient and public involvement (PPI) is recognized as an important component of high‐quality health services research. PPI is integral to the Pre‐hospital Outcomes for Evidence Based Evaluation (PhOEBE) programme. The PPI event described in detail in this article focusses on the process of involving patients and public representatives in identifying, prioritizing and refining a set of outcome measures that can be used to support ambulance service performance measurement.

**Objective:**

To obtain public feedback on little known, complex aspects of ambulance service performance measurement.

**Design:**

The event was codesigned and coproduced with the PhOEBE PPI reference group and PhOEBE research team. The event consisted of brief researcher‐led presentations, group discussions facilitated by the PPI reference group members and electronic voting.

**Setting and participants:**

Data were collected from eighteen patient and public representatives who attended an event venue in Yorkshire.

**Results:**

The results of the PPI event showed that this interactive format and mode of delivery was an effective method to obtain public feedback and produced a clear indication of which ambulance performance measures were most highly favoured by event participants.

**Discussion and conclusions:**

The event highlighted valuable contributions the PPI reference group made to the design process, supporting participant recruitment and facilitation of group discussions. In addition, the positive team working experience of the event proved a catalyst for further improvements in PPI within the PhOEBE project.

## 1 INTRODUCTION

Patient and public involvement (PPI) is recognized as an important component of good‐quality health services research internationally and in the UK is viewed as central to national health research policy by the Department of Health (DH), National Health Service (NHS) and National Institute of Health Research (NIHR)^1^, ^2^, ^3^ The Research Governance Framework (RGF) for Health and Social Care^2^ states that research should be “pursued with the active involvement of service users and carers including, where appropriate those from hard to reach groups” and that patients should be involved at every stage of the research process where appropriate. “Hard to reach groups” (also termed “seldom heard”) may be defined as those from minority or socially disadvantaged groups for example minority ethnic, LGBT (lesbian, gay, bisexual, transgender) or homeless people, people with chronic mental illness, drug users or criminal offenders.^4^

Patients may be involved in a *consultation* role (researchers seek the views of patients and public on key aspects of their research); a *collaborative* role (an on‐going partnership between researchers, patients and the public throughout the research process); or *publicly led* (public and patients design and undertake the research). As most NHS‐related research is publicly funded, patients and public have a right to be involved to help improve the NHS and their own health‐care outcomes and experiences. Thus, patients must move from being “mere users and choosers to being makers and shapers of health services.”^5^

There is a compelling argument that patients offer unique insights and knowledge of a clinical condition or experience of care that researchers may not possess. In this way, patients can help researchers to focus on meaningful and relevant issues, improving the overall quality and credibility of research. There is still considerable debate around the best methods to incorporate PPI into high‐quality research. Here, we describe one way this was attempted.

### 1.1 Aims of this investigation

The aims were (i) to assess whether a coproduced, face‐to‐face PPI prioritization event was an effective method of obtaining public feedback and (ii) to find out whether outcome measures considered by health service professionals in a Delphi study were also important to patient and public representatives.

The focus here was the PPI event design and execution. Andy Irving, the PPI lead for the research team, and the PhOEBE PPI reference group members, who provided direct quotes, were the main authors of this article.

## 2 BACKGROUND

### 2.1 PhOEBE research programme

The Pre‐hospital Outcomes for Evidence Based Evaluation (PhOEBE) project is a 5‐year National Institute for Health Research (NIHR) funded research programme which aimed to develop new ways of measuring the quality, performance and impact of pre‐hospital care provided by ambulance services. PPI played an important part in the programme: PPI representatives were coapplicants and involved with design of the programme which involved two ambulance services, Yorkshire Ambulance Service and East Midlands Ambulance Service NHS Trusts.

A PPI reference group was created at the outset to independently consider the PPI issues relevant to the programme and advise the research team. The PhOEBE PPI reference group had three lay members; two from the Sheffield Emergency Care Forum (SECF) and an expert patient advisor working with Yorkshire Ambulance Service NHS trust, focussing on patient safety and experience.

The long running PhOEBE project has had PPI at its heart from the beginning

(Maggie Marsh, PhOEBE PPI reference group member)

### 2.2 Patient and public involvement in PhOEBE

The PhOEBE PPI group met on a regular basis with a named PPI lead from the PhOEBE research team (Andy Irving, AI), working to an agreed “terms of reference” document (Supporting Information). One PPI representative was also a member of the Project Management Group (PMG) and Study Steering Committee (SSC). This ensured a lay perspective on significant decisions within the project was considered and so acted as a link between the research team and PPI group.

At the beginning of the PhOEBE project, potential ambulance performance and quality measures were identified from two systematic reviews of related policy and evaluation research. These were then prioritized using a three‐stage consensus process: Stage 1 A Multistakeholder consensus event; Stage 2 A Modified Delphi study; Stage 3 A Coproduced PPI event. The details of this a three‐stage multimethod approach are reported separately.^6^ This iterative approach allowed the gradual refinement of a large list of ambulance service quality and performance measures down to a smaller agreed number of indicators for further development reflecting both service provider and public perspectives.

Lay members participated in the Stage 1 consensus event, and the research team had originally intended to also include them in the Delphi study. However, in the initial stages of developing the Delphi questionnaire, the PhOEBE PPI reference group raised concerns about the ease of understanding the complex, technical medical language used and its appropriateness for a lay audience.

We three of the PPI reference group had meetings in 2014 with the research team to reduce the measures further, but I was struggling with the minutiae and the technical language. An impasse came when the research team wanted further results and we were left feeling unsure of the direction we were supposed to go and rather frustrated, as the researchers also seemed to be. We felt that just three of us were a limited number to ask

(Maggie Marsh)

Alternative options were considered for a more user‐friendly questionnaire, containing all the measures alongside lay definitions. The PPI group, considering this too unwieldy and the Delphi method not suited to a lay audience, decided not to pursue or pilot this approach.

I had the inspiration to increase [PPI] to a manageable number, perhaps twenty, of lay people to deliberate, choose and vote on their preferences of the measures in a new consensus day, closely working with the research team to bring this to fruition

(Maggie Marsh)

## 3 METHODS

### 3.1 Codesign phase

The broad aim of the codesign phase was to develop a more interactive way to listen to those who used and cared about ambulance services beyond a mere “tick‐box” exercise while also meeting the requirements of the PhOEBE research programme.

Our specific objectives for the event were for participants to:

1. Understand the work undertaken by the PhOEBE project so far
2. Have an opportunity to discuss performance measures and why they were needed
3. Choose measures which they considered most important
4. Feel they had been involved and their views listened to
5. Understand how the event contributed to the process of selecting ambulance service measures
6. Understand how the measures selected would be used in the next steps of the PhOEBE project

At a series of meetings in March, April, May and June 2014, the PPI reference group and research team members identified several challenges involved in meeting these objectives. At the outset, it was decided that, given these challenges, an external, independent facilitator was needed to coordinate the event, mediate whole group discussions and keep sessions to time. Other key decisions included: a suitable venue; presentation of measures; resources needed; method of registering preferences; organizers’ roles as presenters or discussion facilitators as well as method and target of participant recruitment.

### 3.2 Setting and participants

As everyone was considered a potential patient of the ambulance service, the PPI reference group wanted a representative and diverse sample of participants, ensuring that measures and indicators developed would be relevant, of value and understandable to any patient or members of the public who might wish to interpret them. Efforts were made to invite patients and the public from diverse backgrounds to represent the various potential ambulance service users, particularly those “hard to reach” groups who might not traditionally access such an event.

Participants were recruited through publicizing the event via email letter and flyer to over 20 PPI groups and networks (Supporting Information). The PPI reference group cascaded the invitation via their own networks to other patient and public groups in the Yorkshire, Humber and Lincolnshire areas.

There were no explicit inclusion or exclusion criteria as we wished the event to be accessible and open to all and were fully prepared to make any reasonable adjustments to enable participants to attend and engage. A non‐academic venue, with good transport links, was thought to be the best option; travel reimbursement and a monetary gift in line with INVOLVE good practice were offered to all PPI participants.^7^

### 3.3 Event format

The event was set in an open plan meeting space with four large tables. Each table consisted of around five people with specific roles: three event attendees, a PhOEBE PPI reference group member as discussion facilitator and a research team member on hand to answer any technical queries. To help participants understand the PhOEBE project [objective 1] and be able to discuss the materials presented [objective 3], each table was provided with a resource pack, containing a plain English guide to the measures explaining the concepts and terminology used, and a glossary of the research jargon (Supporting Information). For the purposes of the event and to cover four tables, an additional PPI member from the SECF helped as discussion facilitator.

The idea was for the research team to present how ambulance services work from the initial call; problems they face; what the PhOEBE project is and progress so far; presentation of the measures for consideration by lay people; discussion of measures in small groups; voting individually on preferences; conclusion; feedback on the day and results. Each section was to be about 15 minutes long; using video clips where appropriate and giving time for questions and answers before moving on to the next section. A glossary of technical language in plain English I also considered necessary and wrote it with the help of the research team

(Maggie Marsh)

Mindful of the potentially overwhelming amount of information involved, the PPI reference group felt the day's event should be tightly structured. The day was subdivided into to three main sessions, based on the groups of measures we wanted PPI opinions on: Patient Outcomes, Clinical Management and Whole Service measures. These groups were further subdivided for nine separate voting rounds (See Supporting Information for a full list of measures).

The exact nature and scope of the participation task were clearly described by the independent facilitator at the beginning and checked at regular intervals throughout the event to confirm all participants understood what was expected.

To ensure participants understood the PhOEBE project [objective 1], it was agreed that researchers would initially describe sets of ambulance measures using 10‐ to 15‐minute PowerPoint presentations to the whole group. To further support participants understanding and to promote open discussion, involvement and active listening [objective 2, 4] PhOEBE PPI reference group members would then facilitate 10‐ to 15‐minute discussion within small groups on each table, allowing each event attendee to ask questions and clarify any issues.

**Figure 1:**
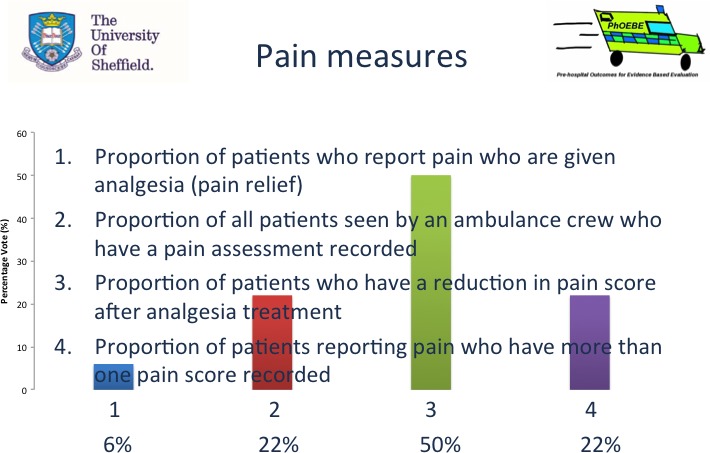
Voting round 1 of 9, the pain measures voting slide

To promote active involvement [objective 4] and register which measures they thought were most important [objective 3], participants were asked to take part in a structured decision‐making process, voting on measures using Turning Technologies© (Turning Technologies, Youngstown, OH, USA).^8^ Turning Technologies is an audience response voting system that enables anonymous voting with the facility to show the audience instant results in the form of a bar chart and percentages overlaid on the slide. Turning Technologies data quality checks verified that all participants voted in all nine voting rounds. To vote on which measure they thought most important in each group, participants (n=14, plus the four PhOEBE PPI members) selected measures corresponding to numbers (1‐9) on a keypad, and results were automatically calculated and presented for each measure as a percentage (see example Figure 1 below).

At the end of the event, a summary of the results from the nine voting rounds was presented. To achieve objective 5 and 6, a final researcher‐led PowerPoint presentation explained how these results would feed forward into the next steps of the PhOEBE project. Finally, participants were also given the opportunity to provide feedback about the event itself both on paper feedback forms and using anonymous voting.

## 4 RESULTS

### 4.1 Key results on the process of the event

Overall 16 individuals registered to attend the event and 14 attended (88%) representing people from three broad participant categories: “hard to reach” groups (n=3), students and aged under 25 years (n=3), and representatives from local and regional patient involvement and advocacy networks (n=8). A full list of event invitees and attendees is presented in an Supporting Information. Participants answered a brief set of evaluation questions at the end of the day using the Turning Technologies voting method to see whether the event had met its objectives. A member of the PPI Reference Group participated in the feedback increasing the numbers from 14 to 15 (Table 1) .

**Table 1:** Participant feedback from votes

Objective	Question	Response (Yes or No)
1	Have you understood what PhOEBE is all about?	Yes, 100%
2	Have an opportunity to discuss performance measures and why they were needed	Yes, 100%
4	Feel they had been involved and their views listened to	Yes, 100%
Extra	Have you enjoyed the day?	Yes, 100%
Extra	Do you think the approach we've used today is a useful model for future PPI events?	Yes, 93%, No, 7%

Feedback via voting at the end of the event confirmed the first four objectives were fully met. Participants were not asked to consider objective 3 as it was evident this objective had been met as votes had recorded participant's views on measures considered most important.

Extra questions confirmed that all participants had enjoyed the day and that 14 of 15 (93%) felt that such an event was a useful model for future PPI work. To give participants further opportunity to give feedback, paper forms were also used (Table 2).

**Table 2:** Feedback from paper forms

Question/rating	1 Strongly disagree	2	3	4	5 Strongly agree
The event gave me an opportunity to learn about this research in sufficient depth			1 (6.7%)	4 (27%)	10 (67%)
I understood the aims and objectives of the event.				5 (33%)	10 (67%)
The aims and objectives of the event were met.			1 (6.7%)	4 (27%)	9 (60%)
This is a good way of getting patients and the public involved in research.			2 (13%)	2 (13%)	11 (73%)
I would attend an event like this again.				3 (20%)	12 (80%)
Overall I enjoyed the event.				3 (20%)	12 (80%)

In “additional comments” boxes, participants also made some very positive statements about the event:

“Good use of voting technology”
“Fascinating group discussion. Very good way of choosing answers. Great level of expertise”
“Good provision of resources”
“Good balance of debate, reflection and voting”
“Aimed at just the right level for me”

Clearly, the vast majority of participants felt they had understood the aims of the day, felt the objectives had been met and enjoyed this method of involvement, specifically that the format and mode of delivery made this an effective method to obtain public feedback.

Participants also raised issues around things that could have been improved

“Difficult to choose some points as ideally you would measure everything”
“More regional spread of the general public”
“Try to spread to youth services, eg young carers”

Comments regarding the difficulty in choosing measures and issues around participant sample are addressed in the discussion.

To fulfil the fifth and sixth objective (*Understand how the event contributed to the process of selecting ambulance service measures, Understand how the measures selected will be used in the next steps of the PhOEBE project*), all attendees received a report of the results and feedback 3 weeks after the event.

There were several costs associated with the development and delivery of this event outlined in Table 3 below.

**Table 3:** Costs

Item	Description	Cost
Independent facilitator	Including x3 codesign phase preparatory meetings, planning and event delivery.	£1900
Event location hire including catering	Large meeting hall, lunch and refreshments for 38 guests.	£1313.16
Participants travel reimbursement	11 of 14 attendees claimed public transport or mileage costs at 40p per mile	£105.40
Participants payment	All 14 participants were paid £50 in cash on the day for participation	£700
Total		£4018.56

### 4.2 Key results on the outcomes of the event

The PPI event produced a clear indication of which measures were most highly favoured by participants (see Table 4 voting results from the PPI event). The highest‐ranking measures are presented according to the percentage of votes achieved (see Table S1, S2 and S3 for a full list of measures and votes).

**Table 4:** Voting results from the patient and public involvement (PPI) event

Voting round	Measures group Delphi score	Highest voted measures	% Vote	Delphi score^a^	Included in final measures
1	Patient outcomes	Proportion of patients with a life‐threatening condition (amenable to emergency treatment) who are discharged alive from hospital.	61	7	Yes
2		Proportion of patients who have a reduction in pain score after analgesia treatment.	50	7	Yes
3		Proportion of all 999 calls recontacting the ambulance service with 24 h	44	7	No
4	Clinical management	Number of calls prioritized correctly to appropriate level of response as a proportion of all 999 calls.	67	8	Yes
5		Proportion of all cases with a specific condition who are treated in accordance with established protocols and guidelines, for example stroke, heart attack, diabetes, falls.	67	8	Yes
6	Whole service	Proportion of emergency calls with a response time within an agreed standard.	78	8	Yes
7		Proportion of eligible patients who arrive at definitive care within agreed timescales.	50	8	No
8		Proportion of category A calls attended by a paramedic.	28	7	No
9		Proportion of patients who are treated on scene or left at home who are referred to an appropriate pathway or primary care.	25	7	No

a Delphi Score ≥8=Good consensus, 6‐7=Moderate consensus, <6 (low)=Low consensus.

Alongside other key considerations, the results of the PPI event guided the research team to select 5 of the 9 most highly voted measures to be included in the final measures for further development.

Table 5 also shows the high degree of agreement between measures considered important by clinicians and academics in the Delphi survey, indicated by a moderate or high consensus score and by PPI via the PPI event votes. There was only disagreement on item 7. Delphi participants rated this with moderate consensus as being a good measure of the quality of care provided by ambulance services while only one PPI event participant in either voting rounds voted for this measure.

**Table 5:** Final list of PhOEBE measures (Delphi and patient and public involvement (PPI) scores)

No.	Measure description	PPI vote (%) and rank within vote category	Delphi score^a^
1	Mean reduction in pain score	50% 1st of 4	7
2	Accuracy and appropriateness of call ID	67% 1st of 4	8
3	Median response time	78% 1st of 3	7
4	Proportion of decisions to leave a patient at scene (hear & treat and see & treat) that were potentially inappropriate	N/A^b^	N/A
5	Proportion of ambulance patients admitted to hospital with a serious emergency condition who survive to 30 d post‐incident	61% 1st of 3	7
6	Proportion of ambulance service contacts for patients with specific, urgent health problems presenting a low risk of death, where the patient subsequently died from such a cause within 30 d	N/A^b^	N/A
7	Proportion of patients transported to ED by 999 emergency ambulance who were discharged to usual place of residence or care of GP, without treatment or investigation(s) that needed hospital facilities	3% 7th of 7 in both rounds.	7
8	Proportion of all cases with a specific condition who are treated in accordance with established protocols and guidelines, for example stroke, heart attack, diabetes, falls	67% 1st of 3	8

a Delphi Score ≥8=Good consensus, 6‐7=Moderate consensus, <6 (low)=Low consensus.

b These measures were formed from related items after the Delphi and PPI event and therefore were not scored or voted on directly.

A full list of Delphi and PPI event results are presented by category of measure (see Tables S1, S2 and S3). A more detailed study methodology and integrated analysis of results are reported in a separate paper.^6^

## 5 DISCUSSION

The PPI event provided a clear indication of measures preferred by event attendees using a format that was considered useful, informative and relevant. It also added value in other ways. The PPI reference group had an opportunity to extend their influence and involvement particularly in relation to participant recruitment, discussion facilitation and content of resource packs provided to event participants. Closer working with the PhOEBE PPI reference group and research team at all stages of the event proved a catalyst for further improvements in PPI in the project. Increased contact and communication with the PPI lead also created closer collaborative relationships between the research team and PPI reference group members that helped support further PPI activities.

Following the success of the event, the PPI reference group were inspired to codesign a poster to share best practice from their experiences. The poster was presented by PPI members at two national conferences (INVOLVE November, 2014 and 999 EMS Research Forum, February 2015). This demonstrated a high level of commitment and willingness to take on new design and dissemination activities. The 999 EMS Research Forum conference abstract was published in the Emergency Medical Journal Online.^9^

There is no doubt that the PPI Reps have been involved and invited to contribute to every stage in the process of bringing this event together. This took some time to grasp initially as there was some concern around being asked to ‘lead’

(Andrea Broadway‐Parkinson, PhOEBE PPI reference group member)

The PhOEBE PPI reference group demonstrated willingness to be “makers and shapers” as research collaborators. This was made possible by mutual respect, commitment and positive attitudes between the research team and PPI reference group, meaning the latter were willing and able to take on this task. Developing trust and teamwork of this nature takes time and resources. Without this, there was a danger that disingenuous attempts to co‐opt members of the public and patients into pseudo‐collaborative roles, while maintaining total control of the research process would only reinforce and replay divisions between researchers and patients.^10^

We can be proud of what has been achieved since [2014] and how things have definitely become more PPI focused and co‐collaborative

(Andrea Broadway‐Parkinson)

Each of the three‐stage consensus process provided a key function: the Multistakeholder consensus event identified key concepts related to ambulance service quality and performance; the Delphi process was used to develop and refine measures related to these concepts; the PPI event then allowed PPI members to engage with and provide an input into the prioritization process.

There are various reasons why measures from the Delphi study and PPI event may or may not have been taken forward for further development. A final subset of PhOEBE measures was derived through consideration of both the Delphi and PPI scores by small expert group discussions. Other factors such as feasibility and availability of data, relevance to ambulance care, whether measures were already being used, and if they related to the whole or part of the ambulance population had to be considered when creating the final set of measures (See Table 5)

The Delphi and PPI disagreement around measure 7 (Table 5, regarding “*the proportion of patients taken to ED without treatment or investigation(s) that needed hospital facilities*”) illustrated an important issue. Delphi participants (academics, managers, commissioners, clinicians) may have been more attuned to the whole service resource implications of potentially inappropriate conveyance decisions and therefore agreed (moderate consensus) on this measures’ utility.

Patient and public involvement event participants in this round of voting, however, favoured “*Proportion of category A calls attended by a paramedic*” (*28% highest voted of 7*) which may indicate a traditional preference for paramedics which does not reflect recent changes in the roles and skills within modern ambulance services. This example highlights the inherent difficulty in choosing between measures as noted by one of our event participants. It also underlines the importance of including a range of stakeholders with different types of knowledge and experience in PhOEBE's multistage study so that patient and public preferences were balanced alongside clinical and systemwide perspectives.

### 5.1 Strengths and limitations

Venue hire, catering, PPI payments for event attendance, travel expenses and the appointment of a paid external independent facilitator in total cost just over £4k. We acknowledge that these are funds that not all projects have. However, as research funders are often proactive in ensuring PPI is properly funded, it is the researchers’ responsibility to appropriately consider and budget for such activities within grant applications. Marsden and Bradburn^11^ recommend that an external facilitator is used in such involvement activity, as being independent of the subject of enquiry may help in developing collaborative working. The experience of the research team, including the PPI reference group, suggested that the external facilitator was a particular strength and helped the PPI and research team to deliver a successful event. Staniszewska et al.^12^ identified adequate financial resources for public involvement in research as being critical for researchers to develop and deliver good‐quality health research with the public.

Bringing in Mark as facilitator to overcome PPI Representative concerns about facilitating and running a PPI event was a great idea! On balance, I think Mark as a facilitator was invaluable to the success of the PPI event and should be costed in at future events

(Andrea Broadway‐Parkinson)

The Phoebe project has had the luxury of time and resources to; hold open days, involve PPI members, create posters and explain them to audiences at conferences. [We have created] content that will make it clear that involvement wasn't box ticking. [We will] finish off with an animated lay summary on the internet to ensure that people can see what the project has worked towards

(Dan Fall, PhOEBE PPI reference group member)

Although there are examples of successful PPI in Delphi surveys,^13^ the PhOEBE PPI reference group raised concerns around the appropriateness of this method for a lay audience in this particular project. Attempts were made to develop a lay version of the Delphi survey to enable participation in the same way as other clinical and academic participants. This proved difficult to do without losing the original meaning of the Delphi questions or making the questionnaire so long that participants would not want to take part.

Given the technical medical language and concepts involved in the PhOEBE Delphi questionnaire, our PPI event method offered greater opportunities for more interactive engagement and personal contact in the process of incorporating user views in to the prioritization process. However, obtaining PPI views using a separate face‐to‐face workshop (rather than a Delphi questionnaire) introduced some limitations. For example, it was not possible to include all measures from the Delphi survey in the PPI workshop. This was due to practical constraints regarding how many measures the PPI participants could feasibly consider during a 1 day event, given that each measure required substantial explanation and group discussion. There were also limitations on the amount of time PPI were able to contribute to the day, as well as travelling distances and potentially complex health problems to consider for participants.

Combined with the challenging/abstract nature of the research topic from a PPI perspective, barriers such as geographical location, start and break times, travel and support needs, etc. need to be more fully understood

(Andrea Broadway‐Parkinson)

Feedback comments from participants reinforced our view that the format, length of the day, sequencing and mixture of researcher presentation and interaction in the event worked well. The use of the Turning Technologies voting and PhOEBE PPI reference group members as discussion facilitators enabled participants to discuss confidently and feel listened to which made this an effective method for obtaining public feedback.

I felt that the day had been successful on many levels. As an educator, I enjoy problem solving and was pleased to have had my ideas taken up and thought useful by the research team. Both sides need to have mutual confidence and trust

(Maggie Marsh)

However, when it comes to working with PPI participants at all stages of the research process, from project design, event coproduction through to writing and dissemination, the use of technology such as electronic voting, emails and word processing software must be carefully considered.

Technology is assumed to be no barrier to PPI involvement however it seems obvious that it must be. Do they all have the same level of understanding required to function in the team? What equipment do they have at home? When attempting to get written PPI content for an academic paper submission, comments were raised where the editors asked for resubmissions all with ‘track changes’ from MS Word. What if participants only have a smart phone or not even that?

(Dan Fall)

There were some limitations to the PPI event reported. Although efforts were made to engage with diverse groups across Yorkshire, including those representing people within the region with sensory impairments or learning difficulties, no participants were successfully recruited. It was assured that presentations and materials would be made available in appropriate formats on the day (eg Easy Read, large print, Braille or audio) but more could have been done to promote the event itself in these formats. However, a key strength of the recruitment process was the use of the PPI reference groups’ own personal contacts and networks beyond local PPI groups known to the research team. As Wilson et al.^14^ found, PPI representatives who act as a link to broader constituencies is an effective PPI model.

The information had been sent out pan Yorkshire and Humber so that a wider catchment of people had an opportunity to hear about PhOEBE. In the future we could consider a ‘Roadshow approach’—to overcome the geographical barriers of hosting only in Sheffield. I am convinced that few people beyond Sheffield turned out because of location and travel barriers

(Andrea Broadway‐Parkinson)

Feedback comments highlighted that young people (aged less than 18 years) were also not represented at the event. This was due to the fact that no specific local or national youth organizations were contacted.

In future PPI events, efforts should be made to consult INVOLVE's “A Guide to Actively Involving Young People in Research”^15^ and make necessary adjustments to the mode and level of engagement for this specific group.

Emergency pre‐hospital care is defined by its short‐term transitory nature. Everyone is a potential user of ambulance services but few people would identify themselves as regular users and those who do may be atypical. This can make involving patient and the public in emergency care research challenging if no one identifies themselves as potential beneficiaries of such research or is willing to speak up on behalf of patients who use emergency care. Groups like the SECF^16^ have enthusiastic and committed members like Maggie and Dan, with wide‐ranging knowledge of pre‐hospital and emergency care who provide critical patient perspectives within research and are not afraid to advocate on behalf of this patient group.^17^

The PPI event benefited from service users of a local addiction treatment service attending. Such groups are typically hard to access and may not ordinarily attend such a research event despite being potential users of the ambulance service and so of direct relevance to them. In this way, as endorsed through the feedback and evaluation process at the event, the added value of our carefully considered PPI friendly methodology served to empower disadvantaged or typically stigmatized groups in society. This was made possible using the researchers’ (AI) contact with local drug and alcohol services and service users, highlighting the value in building good relationships with local community groups.

The primary objective of this event was to obtain feedback from a wider PPI audience on ambulance service performance measurement. We did not set out to “do research” on the PPI participants themselves; therefore, demographic or other data were not collected from the PPI event participants. As a result, the representativeness of the participants in terms of age, gender, disability/impairment, ethnicity, etc. cannot be commented upon. Despite efforts to invite PPI from diverse backgrounds, no claim to have achieved a representative sample can be made.

## 6 CONCLUSION

While there is no single correct method for involvement, there are some key ingredients that researchers and PPI may wish to adopt. The PhOEBE PPI reference group was instrumental in the design and execution of the PPI event but to achieve this took time, patience and teamwork. We should be clear that to deliver such an event also takes significant staff resources. The role of the PPI lead was important in building relationships, developing trust, communicating and in maintaining momentum for involvement within the PhOEBE project. The RAPPORT^14^ PPI evaluation concluded that developing good relationships and having a dedicated PPI coordinator, either internal or external of the team, is significant in providing effective PPI.

In conclusion, this article has presented a method of involvement, which proved effective in obtaining patient and public feedback on complex, little known aspects of ambulance service performance measurement and in building capacity for further PPI within the PhOEBE project.

## CONFLICTS OF INTEREST

None.
