# Supplementary material for: A coproduced patient and public event: An approach to developing and prioritizing ambulance performance measures
Source: Health Expect. 2017 Aug 25;21(1):230–8. doi: 10.1111/hex.12606 (PMC5750774; doi:10.1111/hex.12606)
Supplement: Supplementary file 1 [file HEX-21-230-s001.docx]

Table S1: Delphi and PPI Patient Outcome measures

| **Patient outcomes measures** | **Delphi score** | **Included in PPI** | **PPI vote %** |
| --- | --- | --- | --- |
| Proportion of patients who report pain who are given analgesia (pain relief) | 8.0 | Y | 6% |
| Proportion of patients with cardiac arrest where resuscitation is attempted at the incident scene who have a pulse on arrival at the emergency department | 8.0 | Y | 11% |
| Proportion of all  999 calls referred for telephone advice only re-contacting the ambulance service within 24 hours | 8.0 | Y | 11% |
| Proportion of all patients seen by an ambulance crew who have a pain assessment recorded | 7.0 | Y | 22% |
| Proportion of patients who have a reduction in pain score after analgesia treatment | 7.0 | Y | 50% |
| Proportion of patients reporting pain who have more than one pain score recorded | 7.0 | Y | 22% |
| Proportion of patients who report that key aspects of care were delivered. (examples of key aspects are timeliness of response; reassurance; professionalism; communication; smooth transition between/within services | 7.0 | Y | PPI unable to vote – too broad a measure |
| Proportion of patients with a life-threatening condition (amenable to emergency treatment) who are discharged alive from hospital | 7.0 | Y | 61% |
| As above but for specific clinical conditions (e.g. stroke, heart attack, cardiac arrest) | 7.0 | N | N/A |
| Proportion of all 999 calls re-contacting the ambulance service within 24 hours | 7.0 | Y | 44% |
| Proportion of patients left at home who are admitted to hospital within 72 hours | 7.0 | Y | 11% |
| Proportion of patients who have a wound treated at home who subsequently develop a wound infection | 6.0 | N | N/A |
| Proportion of 999 callers who die within 0 - 48 hours of first call | 6.0 | Y | 28% |
| Proportion of callers who died at different time points: specific groups e.g. condition, demographics, service | 6.0 | N | N/A |
| Proportion of patients left at home who have a contact with any emergency/urgent health service within 24 hours | 6.0 | Y | 33% |
| Proportion of patients left at home who are admitted to hospital within 72 hours | 6.0 | Y | N/A |
| Proportion of patients left at home who have a contact with any emergency/urgent health service within 72 hours | 5.5 | Y | N/A |

Table S2: Clinical Management measures

| **Clinical Management measures** | **Delphi score** | **Included in PPI** | **PPI vote (%)** |
| --- | --- | --- | --- |
| Proportion of all calls referred for telephone advice returned for a 999 ambulance response | 8.0 | Y | 11% |
| Number of calls prioritised correctly to appropriate level of response as a proportion of all 999 calls | 8.0 | Y | 67% |
| Proportion of life-threatening category A calls correctly identified as category A | 8.0 | Y | 17% |
| Proportion of all cases with a specific condition who are treated in accordance with established protocols and guidelines, for example stroke, heart attack, diabetes, falls | 8.0 | Y | 67% |
| Proportion of cases that comply with end of life care plans where these are available | 8.0 | Y | 0% |
| Proportion of all cases with a specific condition who meet established criteria for transfer, who are transported to an appropriate specialist facility, for example a heart attack, stroke or major trauma centre | 8.0 | Y | 33% |
| Proportion of calls for specific condition correctly identified at during the call, e.g. cardiac arrest, stroke, heart attack | 7.0 | Y | 5% |

Table S3: Whole system measures

| **Whole System measures** | **Delphi score** | **Included in PPI** | **PPI vote (%)** |
| --- | --- | --- | --- |
| Proportion of eligible patients who arrive at a major trauma centre within 45 minutes | 9.0 | Y | N/A |
| Time of call to CPR start time (if CPR is required) Average time from call to start of CPR in cases of cardiac arrest | 9.0 | Y | 0 |
| Proportion of eligible calls who arrive at definitive care within agreed timescales e.g. at a specialist heart attack centre within 150 minutes | 9.0 | Y | 50% |
| Proportion of eligible calls who arrive at a specialist stroke centre within 60 minutes | 9.0 | Y | N/A |
| Proportion of emergency calls for conditions that are not life-threatening with a response time of 30 minutes or less | 8.0 | Y | 11% |
| Number of completed patient clinical records as a proportion of all cases attended by the ambulance service in accordance with minimum agreed dataset | 8.0 | N | N/A |
| Proportion of emergency calls with a response time within an agreed standard for calls for life-threatening conditions | 8.0 | Y | 11% |
| Proportion of emergency calls answered within 5 seconds | 8.0 | N | N/A |
| Time of call to time to definitive care | 8.0 | Y | 50% |
| Number of life-threatening (category A) calls not identified as category A as a proportion of all 999 calls | 7.0 | Y | 14% |
| Number of calls that are not life-threatening identified as category A calls as a proportion of all 999 calls | 7.0 | Y | 6% |
| Number of calls transferred for telephone clinical advice assessment that are completed with self-care advice or referral to an appropriate service as a proportion of call calls transferred for clinical advice | 7.0 | N | N |
| Proportion of category A calls attended by a paramedic | 7.0 | Y | 28% |
| Proportion of patients treated on scene or left at home who are referred to appropriate pathways (primary care) | 7.0 | Y | 25% |
| Proportion of patients transported to ED by 999 emergency ambulance and discharged without treatment or investigation(s) that needed hospital facilities | 7.0 | Y | 3% |
| Proportion of patients who potentially could be left at home who are successfully discharged at the scene. | 7.0 | Y | 14% |
| Unit hour utilisation, urban areas (compared to agreed utilisation) | 7.0 | N | N/A |
| Time of call to time of arrival at scene/ Proportion of emergency calls with response times within agreed standards | 7.0 | Y | 78% |
| Proportion of emergency calls for life threatening conditions with a response time of 4 minutes | 6.0 | N | N/A |
| Proportion of emergency calls for life threatening conditions with a response time of less than 4 minutes | 6.0 | N | N/A |
| Proportion of emergency calls for life threatening conditions with a response time of between 4 - 8 minutes | 6.0 | N | N/A |
| Proportion of emergency calls for conditions that are not life-threatening with a response time of 25 minutes or less | 6.0 | N | N/A |
| Proportion of all calls who receive an ambulance response who are not conveyed to hospital/other health facility | 6.0 | N | N/A |
| Proportion of emergency calls with a response time within an agreed standard for non-life-threatening conditions | 6.0 | N | N/A |
